# Introduction to Topic of the Special Issue “Quantum-Chemical Modeling and Design of Chelate and Macrocyclic Metal Complexes 2.0” from Guest Editor

**DOI:** 10.3390/ijms24119597

**Published:** 2023-06-01

**Authors:** Oleg V. Mikhailov

**Affiliations:** Department of Analytical Chemistry, Certification and Quality Management, Kazan National Research Technological University, K. Marx Street 68, 420015 Kazan, Russia; olegmkhlv@gmail.com

The concept of “macrocyclic compounds” combines very different classes of chemicals that contain nine or more atoms in the macrocycle; macroheterocyclic compounds, the macrocycle of which contains at least one heteroatom, are also distinguished among them. Macrocyclic compounds are extremely extensive; they include such unsaturated and conjugated macrocycles as heteroannulenes and their condensed derivatives—heterocirculenes, cyclic oligohetarenes (porphyrin derivatives, oligothiophenes, etc.), as well as non-conjugated (porphyrinogens, calix-hetarenes) and saturated (crown ethers, cryptands, etc.) cyclic systems. Of particular note are the coordination compounds formed by the macrocyclic organic and organoelement ligands and ions of various *p*-, *d*- and *f*-elements, collectively called “macrocyclic metal complexes”. These substances are characterized by the presence of three or more interconnected (jointed) metal chelate cycles, each of which contains at least two atoms that are part of two neighboring metal chelate cycles; in this connection, the term “macrocyclic metal chelates” is widely used for these compounds. Nevertheless, in the general case, to classify a specific metal complex as a macrocyclic metal chelate, it must contain at least three fused metal chelate rings, each of which contains a complexing metal ion; thus, a metal ion must form at least three chemical bonds with the atoms that make up the macrocycle (which can be formed by both the exchange and the donor–acceptor mechanism).

Due to the correspondence of the diameter of the complexing metal ion to the size and shape of the intramolecular cavity and the presence of several binding centers, the formation of macrocyclic metal chelates occurs with high selectivity, and the complexes themselves are much more stable compared to those chelate complexes in which the ligands are linear analogues of the corresponding macrocyclic compounds (this phenomenon is known as the “macrocyclic effect”). To a large extent, this is why macrocyclic metal complexes in general, and metal chelates in particular, have a number of specific properties that the organic and organoelement compounds that are part of them as ligands do not possess; for this reason alone, they are of not only purely academic, but also very significant practical interest, since they have long found applications in various branches of science and technology, from simple dyes to nanotechnology. It should be emphasized that macrocyclic metal chelates play an important role not only in anthropogenic, but also in non-anthropogenic activities; in particular, metal chelates with tetradentate macroheterocyclic ligands—porphyrins and structurally similar corrinoids—are very common in living nature. Due to four donor nitrogen atoms, which form a planar square structure and are rigidly fixed in space, such ligands form very strong complexes with cations of various chemical elements. These include macrocyclic chelates of M (II) ions of 3*d*-elements with various porphyrin derivatives, which are respiratory and coloring components of blood-heme; hemerythrin (red) and chlorocrourin (yellow-green) contain Fe (II) as a complexator; hemocyanin (blue)-Cu (II); and macrocyclic Mg (II) metal chelate with a porphyrin derivative is a coloring agent of green leaves, namely chlorophyll. For the functioning of biological membranes, macrocyclic metal chelates with cyclic polypeptide ligands—ionophores—are important; they are capable of binding and transporting alkali, alkaline earth metal and ammonium cations through membranes, thereby regulating the content of these ions in the extracellular space and inside cells. The list of such examples could go on and on. In this regard, it is worth noting that the first synthesis of metal macrocyclic compounds took place almost 100 years ago (moreover, this happened quite by accident [1,2]), when copper (II) phthalocyanine was discovered, which, due to its exceptional resistance to light and aggressive environmental agents, finds wide practical application as a blue dye in the paint and varnish industry. This compound was obtained as a result of a very specific chemical reaction, “self-assembly”, when at least one of the ligands that make up such a complex is formed from simpler “building blocks”, and this self-assembly itself occurs only in the presence of certain metal ions. (Modern coordination chemistry uses more specific terms for such “self-assembly”, such as “template synthesis”, “template process” or “template reaction”.) In view of all that has been said, it is quite understandable why the physico-chemistry of macrocyclic metal chelates has acquired special significance in modern chemical science.

In principle, variants of the taxonomy of macrocyclic metal complexes are possible: according to the coordination number of the complexing metal ion, according to the number of metal chelate rings that form the macrocycle, and the number of atoms in each of them, according to the denticity of the ligand that is part of the metal chelate, according to the nature of its donor atoms, etc. Let us, however, turn our attention to the systematics that pertain only to macrocyclic metal chelates and are associated with the location of the metal ion relative to other atoms that make up the macrocycle. Within the framework of this taxonomy, two types of metal complexes can be distinguished: those with a closed loop and those with an open loop. In complexes with a closed loop, the metal ion does not participate in the formation of the macrocycle, but is located inside it in a kind of “chelate cage” formed by a macrocyclic ligand containing at least one macrocycle. Metal chelates of this category contain, as a rule, at least four articulated metal chelate rings, as is the case, for example, in complexes of M (II) with porphyrazine of general formula **I** (although, in principle, metal complexes with three such metal rings are also possible; for example, complexes of M (II) with subporphyrazine formula **II**) (Scheme 1).

Characteristically, at the formation of such compounds, the molecular structure of the macrocyclic ligand forming the metal chelate either does not change at all or undergoes only minor changes. In contrast to closed-loop metal chelates, in open-loop metal chelates, the complexing metal atom is included in the macrocycle, as shown in Scheme 2.

Another difference between these two types of metal complexes is that open-loop macrocyclic metal complexes contain *acyclic ligands* in the inner coordination sphere with a specific spatial arrangement of donor atoms, due to which they form chemical bonds with complexing metal ions. The molecules of such ligands (which have even received the special term “compartmental”) are distinguished by a pronounced flexibility, due to which they are capable of very significant distortion of their structure upon coordination with ions of various metals. Theoretically, a hybrid variant is also possible when a macrocyclic metal chelate, within the framework of the above systematics, can be assigned to both the first and second of these categories, but this is possible only if it includes at least two macrocycles and at least two metal ions (which can be either the same or different). Mononuclear macrocyclic metal chelates, however, can be assigned to only one of these two types. By the beginning of the 21st century, the number of publications on the synthesis and study of the physicochemical properties of macrocyclic metal complexes was already in the thousands; at the same time, they included not only original articles and reviews, but also special monographs (see, in particular, refs. [3,4,5,6]). In this connection, it should be noted that the vast majority of these compounds (both open- and closed-loop) contain *d*-metal ions and polydentate organic ligands with donor nitrogen, oxygen and sulfur atoms. Currently, template synthesis is one of the most important synthetic techniques in supramolecular chemistry, where macrocyclic compounds also play a very important role.

To predict both the physicochemical properties of various macrocyclic metal complexes and to control the processes of their preparation in an experiment (within the framework of template synthesis), information on the parameters of their molecular and electronic structures is very important. However, in a number of cases, their determination for such chemical compounds presents very significant difficulties, and therefore it becomes important to predict the above parameters that determine the physicochemical properties of compounds of the above category. Such a problem is currently quite successfully solvable due to the availability of both modern quantum chemical calculation methods and computer technologies and the corresponding experimental equipment.

The existing methods of quantum chemical calculation of chemical compounds in general and complexes in particular can be divided into three groups: ab initio, semi-empirical and density functional methods. The first of these is often referred to as *ab initio* (meaning “from first principles”), and in accordance with this name, these methods do not use any experimental data, nor parameters that are obtained or can be obtained directly from experimental data. In semi-empirical methods, on the contrary, the calculation procedure and the mathematical expressions associated with it include parameters directly borrowed from the experiment or selected in such a way that the calculation of a certain set of so-called “reference” compounds best reproduced the physicochemical properties of these compounds. Density functional methods, which have become widespread in recent decades, occupy an intermediate position, since they also use a number of parameters that approximate the results of theoretical calculations of the properties of the so-called “electronic gas”. These methods, which are based on the idea of the electron density in the ground state, the distribution of which is described by the so-called one-particle Schrödinger equation, are now the most popular in quantum chemical calculations of molecular and electronic structures of chelate and coordination metal complexes of *p*-, *d*- and *f*-elements.

In our opinion, at this point in time, the following three key problems can be distinguished, related in some way to the practical implementation of quantum chemical methods for calculating macrocyclic metal complexes:

🞅 The problem of expanding the assortment of macrocyclic compounds (and, in particular, metal complexes), for which it is possible to carry out quantum chemical calculations. At present, quantum chemical calculations of molecular and electronic structures of a sufficiently high level of reliability (ab initio and DFT methods) for many laboratories/researchers turn out to be possible only for relatively small molecules; the calculation of more complex connections turns out to be very difficult due to the very significant costs of both computational resources and the time for its implementation (which, in some cases, even when using powerful computer technology, can be several months). To a large extent, therefore, the number of theoretical works devoted to quantum chemical calculations of macrocyclic metal complexes with the DFT method, and even more so when using even more advanced quantum chemical methods, is still relatively small in the literature;
🞅 The problem associated with reducing the resource and time costs for quantum chemical calculations, which would allow the use of not only semi-empirical methods and DFT, but also more advanced calculation methods from the ab initio category, the use of which for the calculation of macrocyclic metal complexes at this point, in many cases, is very difficult. This problem, as can be seen, is directly related to the first of the above problems;
🞅 The problem of choosing adequate options used for quantum chemical calculations of macrocyclic metal complexes. For example, within the framework of the currently most popular version, the density functional theory, there are currently at least several dozens of different functionals (both “pure” and, most often, hybrid) that are widely used in the DFT framework for calculating various chemical compounds. These, in particular, are the functionals B3LYP, B3PW91, PBE, M06 and their various modifications. This problem is a direct consequence of the fact that the functionals are not systematically improved, because of which it is impossible to unambiguously predict which functional will lead to a smaller error in the calculation. In general, at this point in time, the state of DFT is such that it is impossible to estimate in advance the error in calculating the molecular and electronic structures of macrocyclic metal complexes using this theory without comparing its results with data obtained using higher levels of theory or with experimental results. Therefore, the choice of an appropriate functional, as well as the basis set, is carried out either on the basis of comparison with data obtained using ab initio methods, or most often on the basis of the researcher’s own experience and published results of DFT calculations by other scientists for similar objects and reactions for their production (and primarily template synthesis). A similar situation occurs in the choice of ab initio methods, as well as semi-empirical methods.

At present, however, quantum chemical calculations using various methods, and primarily DFT, have become an integral part of many studies devoted to macrocyclic compounds in general and macrocyclic metal complexes in particular. These calculations make it possible at the molecular level to study in detail the chemical processes associated with the targeted synthesis and reactivity of these unique compounds, which in turn contributes both to the improvement of methods for their preparation and the creation of substances with predetermined physicochemical properties. Variants of the density functional theory among all modern methods of quantum chemistry have an important advantage in that they provide the optimal ratio between the accuracy/reliability of calculations and the amount of computational resources expended, and largely due to this circumstance, they have become widely used among chemists working in the field of physical chemistry of macrocyclic connections. Additionally, there is no doubt that with the improvement of computer technology, quantum chemical methods will be increasingly used in coordination and organoelement chemistry, and above all, for the calculation of molecules of macrocyclic metal complexes. In view of the foregoing, this Special Issue of the *International Journal of Molecular Sciences* is intended to contribute to progress in this specific area of chemical science.

## Figures and Tables

**Scheme 1:**
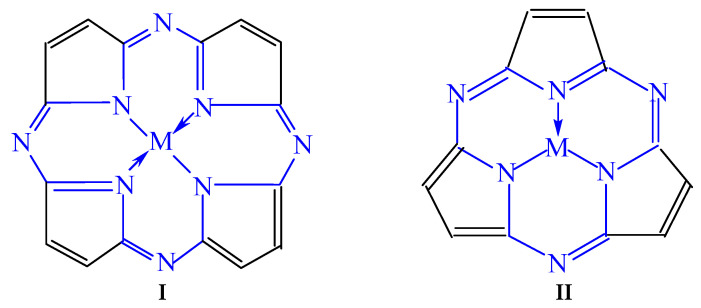
Examples of macrocyclic metal chelates with a closed loop: **I**—with four, **II**—with three articulated metal chelate rings. Metal chelate cycles are highlighted in blue.

**Scheme 2:**
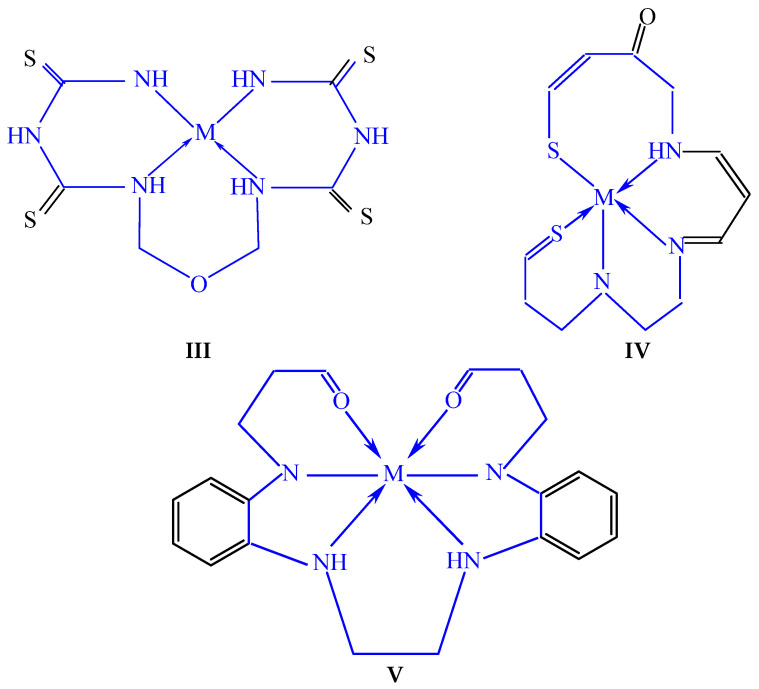
Examples of macrocyclic metal chelates with an open loop: **III**—with three, **IV**—with four and **V**—with five articulated metal chelate rings. Metal chelate cycles are highlighted in blue.
